# Acute Upper Extremity Vein Thrombosis in Recurrent Shoulder Dislocation

**DOI:** 10.7759/cureus.31488

**Published:** 2022-11-14

**Authors:** Abdulaziz A AlRabiah, Atikah T Kadi, Lama I Al Musallam, Albraa A Aldawood, Sulaiman S Alshowihi

**Affiliations:** 1 Emergency Medicine, King Saud University, Riyadh, SAU

**Keywords:** intimal injury, anticoagulation, subclavian vein thrombosis, cephalic vein thrombosis, shoulder dislocation, upper extremity vein thrombosis

## Abstract

The shoulder joint is a commonly dislocated joint in the human body. Shoulder dislocations are commonly associated with fractures of greater tuberosity and neurovascular injuries and are rarely associated with venous complications like deep vein thrombosis (DVT). Ten percent of all venous thrombosis is related to upper extremity venous thrombosis (UE-VT). Severe UE-VT cases are associated with endovenous catheters. Moreover, UE-VT often occurs in specific medical conditions, including hematological malignancy, solid neoplasia, and progressive infection. UE-VT can lead to serious complications such as pulmonary embolism and postthrombotic syndrome. This article presents a case of acute superficial thrombus in the cephalic vein and DVT in the subclavian vein following recurrent shoulder dislocation, which we believe is a rare presentation.

## Introduction

The glenohumeral (i.e., shoulder) joint is one of the most dislocated joints in the human body [1]. Most shoulder dislocations are anterior due to trauma [2]. Despite adequate treatment, numerous patients experience recurrent dislocations [3]. The risk factors for recurrence include males < 30 years of age; playing sports, especially contact or collision sports; a positive apprehension sign; and generalized ligamentous laxity or bone defects [4]. Shoulder dislocations are commonly associated with fractures of greater tuberosity and neurovascular injuries and are rarely associated with venous complications like deep vein thrombosis (DVT) [3].

Upper extremity deep vein thrombosis (UE-DVT) involves a thrombus (i.e., clot) in the radial, ulnar, brachial, axillary, subclavian, internal jugular, or brachiocephalic vein. In contrast, a thrombus in the cephalic or basilic vein indicates upper extremity superficial vein thrombosis (UE-SVT) [5]. Only 4-10% of DVTs occur in the upper extremity. Central venous catheters and underlying malignant disease are the leading causes. Due to UE-DVT, patients are likely to suffer from postthrombotic syndrome, pulmonary embolism (PE), chronic venous insufficiency, thrombophlebitis, a loss of access to the veins, recurrences of the condition, and (rarely) superior vena cava syndrome [6].

Because superficial vein thrombosis (SVT) was once considered a benign and self-limiting condition, it has attracted limited attention clinically and in research. Regardless, recognition that a history of SVT is a significant risk factor for developing venous thromboembolism has increased [7]. SVT most frequently affects the lower extremity and infrequently affects the breast, chest wall, penis, or upper extremity [8]. This article presents a case of acute superficial thrombosis and DVT in the upper limb following recurrent anterior shoulder dislocation.

## Case presentation

In March 2019, a 38-year-old, right-handed Saudi male working as an auto mechanic presented to the emergency department with right upper limb pain, swelling, and numbness for one day. He had complained of bilateral recurrent anterior shoulder dislocation, mainly in the right shoulder, over seven times in the past decade. It usually occurred after playing volleyball or reaching into the back seat of his vehicle while driving.

According to the patient, who rarely remembered how the previous shoulder dislocation happened, the last occurred after a sudden movement of the right shoulder. As usual, he had reduced the dislocation himself at home by lying on the floor in a lateral position and trying to move the shoulder backward and forward until it returns to its neutral position. He presented to the emergency department two weeks after the last shoulder dislocation.

The patient has a history of diabetes mellitus (DM) type two on an oral hypoglycemic agent (metformin), vitiligo, smoking for almost 15 years, and lateral left ankle ligament reconstruction. He denied any history of fever, shortness of breath, chest pain, trauma, falls, or right upper limb surgery.

**Figure 1:**
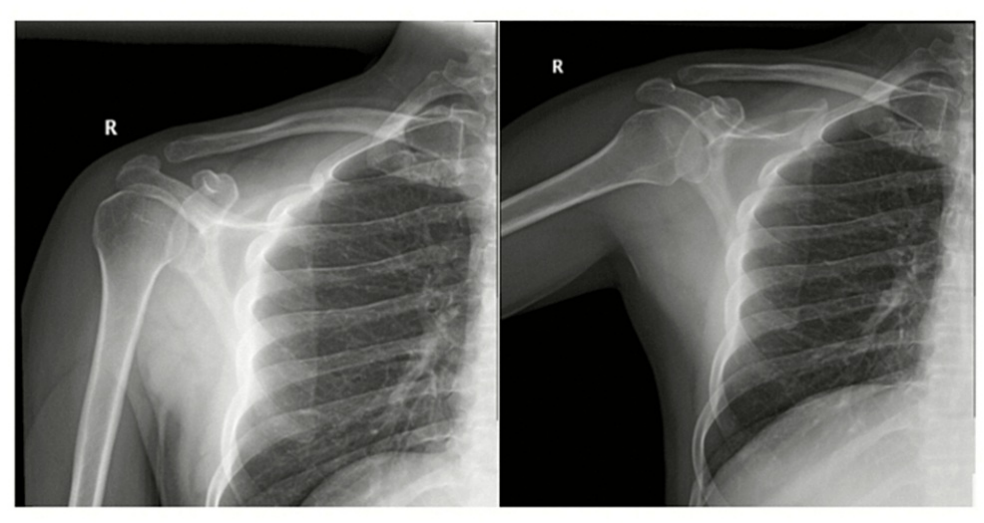
Normal configuration of the glenohumeral joint and acromioclavicular joint with no signs of dislocation or fracture

On physical examination, the patient’s right shoulder was tender and swollen with difficulty raising his arm due to pain. A bedside ultrasound and a shoulder X-ray were ordered in the emergency department as part of his assessment (Figure 1). The right shoulder X-ray appeared normal, with no signs of dislocation.

**Figure 2:**
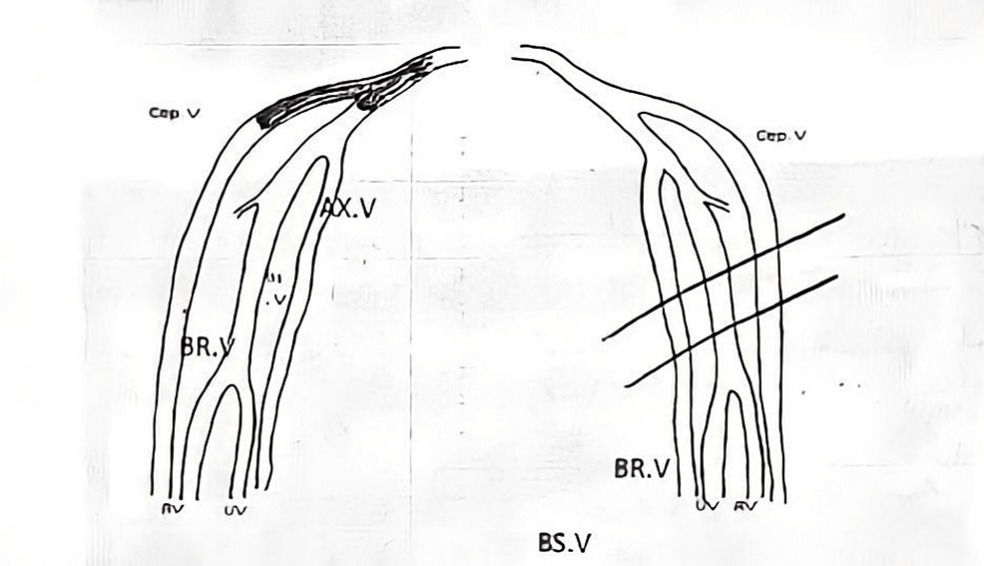
No flow and augmentation of the right subclavian and cephalic veins, indicating acute DVT in the right subclavian vein and acute thrombus in the right cephalic vein Cep.V: Cephalic vein. AX.V: Axillary vein. BR.V: Brachial vein. UV: Ulnar vein. RV: Radial vein. BS.V: Basilic vein

Since the bedside ultrasound revealed no signs of thrombosis or soft tissue infection, a color-flow Doppler (Figure 2) was planned for the following day, and the patient was advised to start taking aspirin, but he forgot to take it. The following day, his symptoms increased; the color-flow Doppler confirmed acute DVT in the right subclavian vein and acute thrombus in the right cephalic vein.

The patient was hospitalized for 24 hours and started on rivaroxaban 15 mg bid for 21 days; then, he took 20 mg for three months and was advised to raise his right hand while sitting and sleeping. The patient underwent blood work (Tables 1-3) to rule out possible risk factors for vein thrombosis.

**Table 1:** Complete blood count and routine chemistry a WBC: White blood cells. b RBC: Red blood cells. c Hgb: Hemoglobin. d Hct: Hematocrit. e MPV: Mean platelet volume. f Hgb A1c: Hemoglobin A1c

Test	Results	Normal Range
WBC^a^	12.6	4–11 x 10^9/L
RBC^b^	5.1	4.7–6.1 x 10^12/L
Hgb^c^	159	130–180 gm/l
Hct^d^	45.6	42–520%
Platelets	220	140–450 x 10^9/L
MPV^e^	8.0	7.2–11.1 fl
Hgb A1c^f^	11.7	4.0–6.0%

**Table 2:** Coagulation a PT: Prothrombin time. b INR: International normalized ratio. c APTT: Partial thromboplastin time. d APCR: Activated protein C resistance

Test	Results	Normal Range
PT^a^	13.80	12–15.5 seconds
INR^b^	1.04	0.80–1.30 seconds
APTT^c^	35	25.70–39.50 seconds
Antithromb III	99	80–120%
Protein C Tot	79	70–130%
Protein S Tot	65	77–143%
APCR^d^	166.6	≥ 120 seconds

**Table 3:** Molecular genetics a JAK2 Mutation: Janus kinase 2 gene mutation. b Factor V Leiden PCR: polymerase chain reaction. c FII Mutation: Factor II mutation. d FII Interp: Factor II interpretation. e FV Interp: Factor V interpretation. f JAK2 Interp: Janus kinase 2 gene interpretation

Test	Results	Normal Range
JAK2 Mutation^a^	Negative	Negative
Factor V Leiden PCR^b^	Negative	Negative
FII Mutation^c^	Negative	Negative
FII Interp^d^	Negative	Negative
FV Interp^e^	Negative	Negative
JAK2 Interp^f^	Negative	Negative

Laboratory studies revealed a high WBC count of 12.6 and a normal hemoglobin level and platelet count. A differential WBC count showed high neutrophils (7.8%), monocytes (1%), and normal lymphocytes. Coagulation profiles were within the normal range, with a partial thromboplastin time of 35 sec, a prothrombin time of 13.8 sec, an international normalized ratio of 1.04, an antithrombin III level of 99, and a protein C level of 79. However, the protein S level was low at 65, the glucose blood level and Hgb A1C were high at 14.1 and 11.7, respectively, and the molecular genetics were all negative. Three years later, he failed to follow up with the hospital. According to him, he had completed the anticoagulant course and had not experienced any similar symptoms.

## Discussion

UE-VT accounts for 10% of all venous thrombosis [9]. While UE-SVT and UE-DVT have almost the same clinical manifestation, UE-DVT is more likely to be associated with fever, whereas UE-SVT is more likely to present with pain and erythema. Both present with edema and can be asymptomatic, especially UE-DVT [10].

UE-VT commonly develops in specific clinical conditions, including hematological malignancy, solid neoplasia, and progressive infection. Furthermore, most cases are associated with endovenous devices, mainly PICC lines [10,11], which is clinically significant because it can cause PE and postthrombotic syndrome [12,13]. Nevertheless, PE rarely complicates UE-VT (4% of UE-DVT and 0.9% of UE-SVT cases) [10].

Before definitive treatment for UE-DVT begins, image diagnosis should be performed using noninvasive methods, such as compression ultrasonography, color Doppler ultrasonography, MRI, and CT, or invasive methods, such as venography, the gold standard for diagnosis. Nonetheless, compression ultrasonography is commonly used to diagnose UE-DVT and eliminates the need for invasive and more expensive tests in most cases [13].

The American College of Chest Physicians recommends managing UE-DVT as follows: In patients with acute UE-DVT that involves the axillary vein or more proximal veins, acute treatment with parenteral anticoagulation (low-molecular-weight heparin (LMWH), fondaparinux, IV UFH, or SC UFH) is superior to no such treatment. Furthermore, LMWH and fondaparinux are recommended over IV UFH and SC UFH [14].

For patients with spontaneous superficial vein thrombosis, the American College of Chest Physicians suggests prophylactic or intermediate doses of LMWH or intermediate doses of UFH for at least four weeks. Oral nonsteroidal anti-inflammatory drugs should not be used with anticoagulation. Medical treatment with anticoagulants is recommended over surgical treatment [15]. Two mechanisms can explain the coexistence of superficial and deep vein thrombosis: 1) the thrombus migrates into the deep venous system, and 2) the hypercoagulable state may clarify the collateral concomitant of the two thrombosis types [16].

It remains unclear what is responsible for the superficial and deep vein thrombosis of the upper extremities in this case report. Nevertheless, we can hypothesize about their pathogenesis. It is possible that the endothelium was damaged by the dislocation and reduction of the shoulder since the patient reduced his shoulder at home [17]. It is theoretically possible for venous thrombosis to develop due to intimal injury combined with poor glycemic control and low fibrinolytic capacity. Specific laboratory tests were performed and demonstrated a protein S level of 65, which is low. Blood glucose and Hgb A1C levels were 14.1 and 11.7, respectively, which is high.

UE-DVT is associated with multiple risk factors mentioned in the literature review. A retrospective cohort study in Nantes showed that, in almost all other cases, venous thrombosis occurred in the specific clinical setting of solid cancers or hematological diseases [10]. In our case, poor glycemic control and protein S deficiency were the only risk factors. In New York, there was a case report of left shoulder and breast swelling brought on by cephalic vein compression and distal basilic vein thrombosis with extension into the axillary and subclavian veins. According to the author, degenerative arthritis was the patient's sole risk factor [18]. A CT scan of a 15-year-old kid who was knocked to the ground and landed on his left shoulder revealed posterior sternoclavicular joint dislocation with left jugular and subclavian vein thrombosis, according to a second case report [19]. Only one case of UE-DVT following anterior shoulder dislocation has been reported. Nevertheless, the patient had a history of falling on his right shoulder [20]. Due to the unusual nature of the risk variables, this case constitutes a singular event of UE-DVT compared to others described in the literature. Therefore, we have described what we think is a unique presentation.

## Conclusions

UE-VT is uncommon and rarely follows shoulder dislocation. It may present asymptomatic or clinical manifestations, including pain, erythema, edema, and fever. UE-VT can also cause major complications such as PE and postthrombotic syndrome. Early diagnosis and treatment can prevent these complications. Doppler ultrasound and venography should be performed immediately if UE-VT is suspected after shoulder dislocation.
